# Controversies concerning thymus-derived regulatory T cells: fundamental issues and a new perspective

**DOI:** 10.1038/icb.2015.65

**Published:** 2015-07-28

**Authors:** Masahiro Ono, Reiko J Tanaka

**Affiliations:** 1 Department of Life Sciences, Faculty of Natural Sciences, Imperial College London, London, UK; 2 Immunobiology Section, Institute of Child Health, University College London, London, UK; 3 Department of Bioengineering, Imperial College London, London, UK

## Abstract

Thymus-derived regulatory T cells (Tregs) are considered to be a distinct T-cell lineage that is genetically programmed and specialised for immunosuppression. This perspective is based on the key evidence that CD25^+^ Tregs emigrate to neonatal spleen a few days later than other T cells and that thymectomy of 3-day-old mice depletes Tregs only, causing autoimmune diseases. Although widely believed, the evidence has never been reproduced as originally reported, and some studies indicate that Tregs exist in neonates. Thus we examine the consequences of the controversial evidence, revisit the fundamental issues of Tregs and thereby reveal the overlooked relationship of T-cell activation and Foxp3-mediated control of the T-cell system. Here we provide a new model of Tregs and Foxp3, a feedback control perspective, which views Tregs as a component of the system that controls T-cell activation, rather than as a distinct genetically programmed lineage. This perspective provides new insights into the roles of self-reactivity, T cell–antigen-presenting cell interaction and T-cell activation in Foxp3-mediated immune regulation.

## Discovery of immunosuppressive T cells

T cells not only induce immune response using cytokines and surface molecules but can also suppress it.^1, 2, 3, 4^ T-cell-mediated immunosuppression was discovered soon after the discovery of thymus as a component of the immune system.^1^ Previous studies had identified immunosuppressive activity in CD8 T cells that were designated suppressor T cells.^1^ Although >4500 papers were published, the area collapsed in the 1980s largely owing to the absence of the ‘suppressor gene', the *I-J* gene, that had been believed to track the suppressor T-cell population.^5^ In the 1990s, the concept of T-cell-mediated suppression revived through the characterisation of suppressive CD4 T-cell populations by two experimental systems: (1) induction of autoimmunity by neonatal thymectomy; and (2) transfer of T-cell populations depleted of specific cell types into lymphopenic mice.^3, 6^ These studies identified CD5^high^, CD25^+^ and CD45RB^low^ as the makers of the immunosuppressive T-cell population and designated these cells as regulatory T cells (Tregs).^2, 3^ Later, the discovery of Foxp3 as a definitive marker of Tregs facilitated the investigation of this T-cell population at molecular and genomic levels.^4^ Currently, it is accepted that some self-reactive thymic T cells escape negative selection and express Foxp3 to become thymic Tregs (tTregs), which suppress self-reactive T cells in the periphery, and thus prevent autoimmunity and maintain immunological tolerance.^2, 3, 4^

## The controversial evidence of neonatal Tregs

### Neonatal thymectomy as the key evidence of tTregs

Originally, Nishizuka and Sakakura^7^ found that thymectomy of 3-day-old neonatal mice induced T-cell-mediated autoimmunity in the ovary and testis, while thymectomy of mice >7 days old did not do so.^7^ The authors hypothesised that helper (Th) T cells are already matured in 3-day-old mice, while suppressor T cells, which are responsible for preventing autoimmunity, are absent in these mice.^8^ In fact, the concept of Tregs gained wide acceptance after the group of Sakaguchi reported that CD25^+^CD4^+^ T cells did not appear in the periphery (spleen) until 3 days of life, while CD25^−^CD4^+^ T cells were already present in the spleen of 3-day-old mice, and transfer of CD25^+^CD4^+^ T cells prevented thymectomy-induced autoimmunity,^9^ thus fulfilling the prediction of Nishizuka.^8^ The finding that thymectomy selectively depleted suppressive CD25^+^CD4^+^ T cells while leaving autoreactive CD25^−^CD4^+^ T cells present^3, 9^ established the view of CD4^+^ T cells that divides them into suppressor and effector cells, thus bridging classical T-cell-mediated suppression and modern Treg biology.^2, 3, 6, 10, 11, 12^

### Tregs exist in neonates

However, several groups found evidence contradicting Asano *et al.*^9^ Suri-Payer *et al.*^13^ found around 10% of CD4^+^CD8^−^ (and/or CD3^bright^) lymph node cells from 2-day-old mice express CD25, and the percentage is identical from 2 days to 6 weeks. Later, Dujardin *et al.*^14^ showed that around 5% of CD4^+^TCRαβ^+^ T cells express CD25 in the spleen of 3-day-old mice on a BALB/c background. More recently, Samy *et al.*^15^ and Monteiro *et al.*^16^ showed that around 5% of T cells from the lymph nodes of 3-day-old mice express Foxp3, using Foxp3-GFP (green fluorescent protein) knock-in mice or anti-Foxp3 antibody, respectively, and that the percentage increased from day 3 to day 5, reaching to 6–10%. All these findings argue against delayed appearance of Tregs in the periphery. Using Foxp3-GFP knock-in mice, Fontenot *et al.*^17^ found that GFP^+^ thymic CD4 single-positive (SP) cells, which are to be tTregs, rapidly increase after birth, from ~0% of CD4-SP on day 1 to ~4% on day 21, but the percentages of CD25-expressing cells in CD4-SP do not differ significantly between neonatal and adult mice (both 4–5%).^17^ Comparable data for peripheral T cells were not reported in the study. The report^17^ has nevertheless been cited as evidence of delayed appearance of Tregs in the periphery,^11, 18^ thus further confusing the issues of Tregs in neonates and thymectomy. Certainly, as thymectomy halts outflow, only T cells in the periphery at the time of thymectomy contribute to the development of autoimmune disease.

Thus, contrary to the currently accepted opinion, the peripheral immune system of 3-day-old mice harbours a significant proportion of Tregs, at least half of that of adult mice. This difference between neonates and adults is much smaller than is widely believed and therefore unlikely to explain the development of autoimmunity following day-3 thymectomy, considering particularly that transfer of <50% of the physiological number of CD25^+^ T cells into adult mice can fully suppress the development of autoimmune disease induced by CD25^−^ T cells.^19^

### Consequences of the controversial evidence

Although there are a pile of molecular data on Foxp3,^4^ the confusion over neonatal Treg development is still a modern problem. It has led to a misunderstanding of the dynamic regulation of CD25 and Foxp3 in neonates and a dismissal of the developmental and regulatory relationship between activated T cells and Tregs. On the contrary to the common belief that is based on the controversial evidence,^2, 3, 10^ CD25 expression in fact occurs at the same time when CD4^+^ T cells are generated and matured in the thymus.^14, 15, 16^ This strongly suggests that CD25 expression is a part of an essential mechanism of CD4^+^ T-cell development, rather than a unique feature of specialised cells. In fact, CD25 is not only a Treg marker but also an activation marker, and Tregs are similar to activated T cells, apart from their Foxp3 expression and poor production of effector cytokines.^2, 3, 10^ Thus the development and the phenotype of Tregs are inherently interrelated with T-cell activation, which is a highly dynamic process. The current dogma, however, over-relies on the expression of a set of transcription factors and their stability and dismisses the dynamic regulations of T-cell response, which always accompanies T-cell activation and subsequent differentiation. It is hoped to obtain a dynamical systems view on how the T-cell system is controlled by these processes, in order to fully understand Treg-mediated regulation.

## An alternative model of Tregs and Foxp3: a feedback control perspective

Accordingly, we propose an alternative view on Tregs and Foxp3, a feedback control perspective, which views Tregs as a component of the system that controls T-cell activation, as opposed to the current dogma, the lineage perspective, which considers that the suppressive mechanism is retained in a distinct lineage of T cells or Tregs.

**Figure 1:**
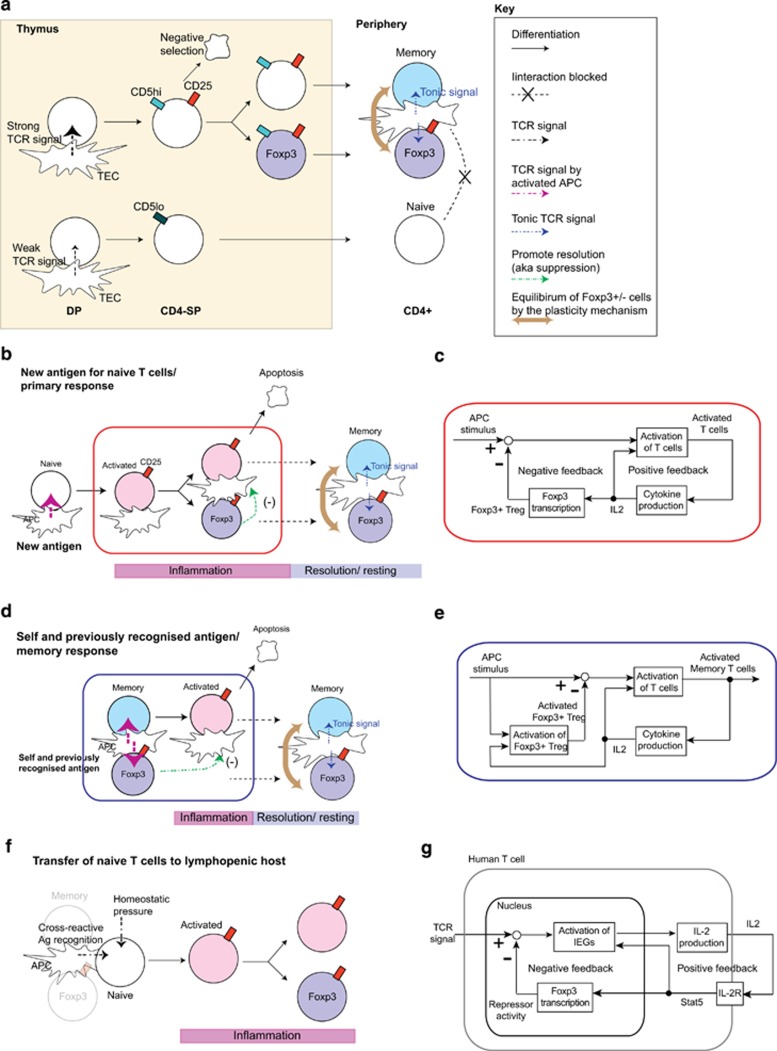
Feedback control perspective of T-cell regulation. (**a**) The proposed model for the T-cell regulation is depicted. Self-reactive thymic CD4-SP receive strong TCR signals from antigen/MHC complex on thymic epithelial cells (TEC) and other APCs. These cells may die by negative selection or survive by expressing CD25 and upregulating the expression of CD5 (CD5^high^), which is a negative regulator of TCR signaling. Some CD25^+^ CD4-SP express Foxp3 and become Foxp3^+^ Tregs in the periphery, while some of the others, we argue, become Foxp3^−^ memory-like T cells (memory). On the other hand, thymic T cells with less self-reactive TCRs may receive weak TCR signals only, remain CD5^low^, do not express CD25 and become naive T cells in the periphery. Thus both Tregs and memory-like T cells are more self-reactive than naive T cells and therefore interact more frequently with APCs that present the same or similar self-antigens in the periphery, receiving tonic TCR signaling, and protecting the antigenic niche from naive T cells. (**b**) Upon encountering with a totally new antigen to the immune system, only some of the naive T cells can respond to the antigen and become CD25^+^ activated T cells. Many of these activated T cells die by apoptosis (that is, activation-induced cell death), but some differentiate into Tregs, promoting the resolution of the response (negative feedback control), and others may become memory T cells, after the resolution. Thus a new antigenic niche is created and occupied by both Tregs and memory T cells, which are maintained by tonic TCR signal in the same manner as for thymus-derived, self-reactive T cells. (**c**) Control mechanism of naive T cells to new antigens shown in panel (**b**). (**d**) Upon encountering with self-antigen or similar antigen on activated APC, both memory(-like) T cells and Tregs immediately respond and are activated. Thus the response will be rapid but be resolved earlier because of the negative feedback by the preexisting Tregs. (**e**) Control mechanism of memory-like T cells and Tregs to self-antigens or experienced antigens shown in panel (**d**). (**f**) Various ‘Treg-depletion' experiments in fact provide to naive T cells an access to all antigenic niches. This effect is most dramatic when both Tregs and memory-like T cells are depleted, and naive T cells have the full access to all niches. (**g**) Possible mechanism for the control of T-cell activation by transient FOXP3 expression in human activated T cells. TCR signal induces and activates immediate early genes (IEGs), which transcribe the *IL2* gene. IL-2 protein is secreted and received by those activated T cells in an autocrine manner. IL-2 signal via IL-2R (including CD25) activates STAT5, which positively regulate the activation of IEGs and FOXP3 transcription. FOXP3 represses IEGs by physically interacting with them or repressing their transcription.

The feedback control perspective is composed of the following three major hypotheses: (1) When an antigen niche (that is, a whole set of antigen-presenting cells (APCs) that present a particular antigen on class II major histocompatibility complex (MHC)) is available, whether in the thymus or in the periphery, T cells with high-affinity T-cell receptors (TCRs) for the antigen interact with the APCs and thus fill the niche; (2) Upon interacting with the cognitive antigen, T cells are activated, producing CD25^+^ activated T cells (or CD25^+^CD4-SP in the thymus). These T cells later generate Foxp3^+^ T cells (Tregs) and Foxp3^−^ memory(-like) T cells (Box 1); (3) Memory-like T cells and Tregs always compete for binding to APCs in each antigenic niche in the periphery and thereby protect the niche from naive T cells, which have low-yet-significant affinity TCRs to the antigen (Figure 1a).

This control mechanism should produce two different types of T-cell response in the periphery. New antigens, to which existing Tregs/memory-like T cells do not have significant cross-reactivity, activate naive T cells only. The naive-derived activated T cells act as positive regulators via a self-amplification of activated T cells (by, for example, autocrine interleukin (IL)-2 and IL-2R), while some of them later express Foxp3 and act as a delayed negative regulator by promoting the resolution of T-cell activation (Figure 1b). Because of the delay in the negative regulation, the system exhibits a prolonged T-cell response (Figure 1c). On the other hand, self-antigens and previously recognised antigens can induce an immediate response of both memory-like T cells and Tregs (Figure 1d). Thus such antigens should produce a faster and shorter T-cell response, because both positive (activated memory-like T cells) and negative (Tregs) regulators are triggered immediately (Figure 1e). Although the significance of implementing these two types of T-cell response of different speeds in response to the two types of antigens is yet to be revealed by a combination of experiments and mathematical modelling, we expect that these two mechanisms are indispensable for the antigen-specific response of CD4 T cells, while tolerating self-antigens, as we discuss below.

## Revisit key evidence of Treg-mediated immune regulation

We may now critically review and examine the key evidence of Treg-mediated tolerance, propose novel hypotheses from the feedback control perspective and suggest critical experiments to address those hypotheses.

### The self-reactive T cells: Tregs and memory-like T cells

The lineage perspective considers that some of self-reactive T cells escape negative selection in the thymus and are selected as tTregs to control self-reactive naive T cells in the periphery.^2, 3, 4^ The feedback control perspective hypothesises, based on a range of evidence discussed below, that naturally arising memory-like T cells partially originate from those self-reactive T cells in the CD25^+^ CD4-SP fraction in the thymus, which is currently considered as the source of Tregs (Figure 1a).

The major evidence that supports this hypothesis comes from transgenic (Tg) TCR studies. It is well-known that T cells with a Tg TCR more preferentially become CD25^+^ Tregs in the presence of a defined (Tg) cognitive peptide.^20^ Furthermore, DO11.10 TCR Tg, *Rag2*^*−/−*^ mice do not develop CD25^+^CD4^+^ T cells^21^ (which in fact include both Foxp3^+^ and Foxp3^−^ T cells; see below), and thus Treg development requires the recombination of the endogenous TCRα for their development, which supports that Tregs develop only when they interact with cognitive antigens. Notably, however, DO11.10 TCR Tg, Rag2^*−/−*^ mice do not develop CD45RB^low^CD44^high^ memory-like T cells either,^22^ the significance of which has not been addressed to date.

The interaction between T cells and antigen–MHC complexes may be the most important determinant for the generation of Tregs (and probably also the memory-like T-cell population). The absolute number, not the percentage, of each Foxp3^+^ Treg clone had an upper limit (at the order of 10^4^) by a bone marrow chimera study using various ratios of wild-type T cells and T cells from a TCR Tg strain expressing a Treg TCR.^23^ In addition, lower chimerism of Treg TCR Tg cells induced higher Nr4a1 expression using a Nr4a1-GFP reporter Tg strain, whose GFP expression reflects the strength of TCR signal.^24^ Each antigenic niche may have a limited capacity that supports those self-reactive T cells, including both Tregs and memory-like T cells, which is experimentally testable using bone marrow chimeras of various TCR Tg.

Tg reporter studies have provided another line of evidence for the self-reactivity of Tregs. Using a Foxp3 GFP knock-in mouse strain with either a null allele or a truncated and dysfunctional protein, GFP^+^ cells, which lack the expression of functional Foxp3 protein, showed a CD25^+ or−^ CD45RB^low^ Treg-like or activated/memory-like phenotype without suppressive activity.^25, 26^ This suggests that their TCRs were self-reactive sufficient to induce and maintain the activated phenotype.

Repertoire studies, however, have not been conclusive to the self-reactivity of tTregs. Some studies showed that Foxp3^+^CD4^+^ T cells had a different TCR repertoire from Foxp3^−^CD4^+^ T cells,^27, 28, 29^ while others showed that TCR repertoires of Tregs and naive T cells were overlapping^30^ and that the same peptide selected both Foxp3^+^CD4^+^ T cells and Foxp3^−^CD4^+^ T cells.^31^ Similarly, repertoire studies only compared the bulk Foxp3^+^ and Foxp3^−^CD4^+^ T cells and dismissed to analyse Foxp3^−^CD4^+^ memory-like T cells, which presumably experienced cognitive antigens. Thus the observed difference of TCR repertoire between the bulk Foxp3^+^ and Foxp3^−^CD4^+^ T cells^27, 28, 29^ may rather reflect the difference between antigen-recognised and non-recognised T cells, as naive T cells dominate the Foxp3^−^CD4^+^ T-cell fraction. It will be necessary to compare the TCR repertoires of Foxp3^+^ Tregs, Foxp3^−^ memory-like T cells and naive CD4^+^ T cells.

### Tonic TCR signalling and Tregs

The original tonic-signalling hypothesis assumes that memory T cells maintain their reactivity by constantly interacting with antigen–MHC complexes in the periphery.^32^ This continuous ‘subthreshold' recognition of self-peptide−MHC complexes is thought to result in a basal activation state that enables T cells to rapidly respond to foreign antigen^33^ and possibly ‘tuning' T-cell responsiveness to self-antigens.^34^ Klein *et al.*^33^ recently proposed that self-reactive thymic T cells are selected as CD5^high^ T cells and receive tonic TCR signalling in the periphery. Interestingly, self-reactive thymic T cells increase the expression of CD5,^33^ which is a negative regulator of TCR signaling,^35^ while the high expression of CD5 is a classical marker of a Treg population.^2, 3^ In agreement with these findings, the conditional knockout study of TCRα showed that Tregs received tonic TCR signalling.^36^

Using an Nr4a1-GFP Tg reporter, Tregs showed a higher level of GFP expression than CD25^−^ T cells in the thymus and in the periphery.^24^ Importantly, the thymic ‘pre-Treg' population, CD25^+^Foxp3^−^ CD4SP, which we argue include the progenitors of naturally arising memory-like T cells, had an even higher level of GFP expression than thymic Foxp3^+^ CD4-SP.^24^ Intriguingly, we have found that CD45RB^low^ memory-like T cells in the periphery also had higher GFP expression using the same Nr4a1-GFP reporter Tg strain (unpublished observation).

Reconciling all the evidence, our feedback control perspective hypothesises that both Foxp3^+^ Tregs and memory-like T cells frequently interact with and compete for antigen–MHC complexes in the periphery, receiving weak yet frequent signal, tonic TCR signal, and thereby maintain their activated/memory-like phenotypes and numbers in each antigenic niche. Then the ratio of the numbers of Tregs and memory-like T cells in each antigen niche would be optimally balanced by their plastic Foxp3 transcription, which may be an alternative explanation for the observed ‘plasticity' of Tregs.^22, 37^ This plastic Foxp3 transcription may be dependent on transforming growth factor-β (TGF-β) and IL-2 and Foxp3-conserved noncoding sequence-1.^38^ These considerations lead to two experimentally testable hypotheses: (1) both Tregs and naturally arising memory-like T cells are lost upon the disruption of tonic signalling, which may lead to the activation of naive T cells; and (2) foreign antigens can differentiate a naive T-cell repertoire into Foxp3^+^ Tregs and memory-like T cells, which persist and receive tonic signal as long as the antigen is available.

### Re-interpretation of Treg-depletion experiments

A series of cell transfer experiments provided an important line of evidence, in addition to the neonetal ontogeny experiments, to establish the current concept of Tregs.^2, 3, 10^ Here T-cell suspensions that were depleted of a T-cell population were transferred into lymphopenic mice, which were analysed for the development of autoimmune and/or inflammatory diseases. The depletion of CD25^+^ T cells induced autoimmune gastritis in nude recipients,^3^ while that of CD45RB^low^ T cells resulted in colitis in *Rag*-deficient or SCID recipients.^10^ Later, Foxp3 expression was found better correlated with CD25^+^ than CD45RB^low^, establishing Tregs as CD25^+^Foxp3^+^ T cells.^2, 3^

Although the lineage perspective tells that self-reactive T cells induce autoimmunity,^1, 2, 3, 4^ this is in fact controversial. Using a Foxp3-GFP reporter Tg mouse in a Foxp3 mutant *scurfy* background, Lahl *et al.*^39^ showed that GFP^+^ cells, which have an active Foxp3 transcription without functional Foxp3 protein and thus considered self-reactive, do not induce autoimmune inflammation when transferred into *Rag1*^*−/−*^ mice. CD45RB^high^ CD4^+^ naive T cells, which are presumably selected as non-self-reactive T cells in the thymus, efficiently induce colitis in recipients, while CD25^−^CD45RB^low^ CD4^+^ T cells, which are composed of memory-like T cells and a small amount of Tregs, do not.^40^ Ono *et al.*^41^ showed that the depletion of GITR^high^ T cells resulted in a more aggressive autoimmune disease with wider organ involvements than that of CD25^+^ T cells by further reducing Foxp3^+^ T cells (~2% vs ~6%). The suppressive activity in CD45RB^low^CD25^−^ or GITR^high^CD25^−^ T cells may be attributable to the small amount of Foxp3^+^ Tregs in the fraction.^4^ However, it may be more straightforward to assume that both Tregs and memory-like T cells are engaged in maintaining immunological tolerance through their high affinity to self-antigens and the plastic Foxp3 transcription. In fact, memory-like CD25^−^CD45RB^low^CD4^+^ T cells can generate Foxp3^+^ T cells more rapidly than CD25^−^CD45RB^high^CD4^+^ naive T cells,^22^ and the memory-like T-cell population in fact contains ex-Foxp3^+^ Tregs by a genetic fate-mapping experiment.^37^

Thus the feedback control perspective hypothesises that self-reactive T cells, which include both Tregs and memory-like T cells, frequently interact and thereby protect the self-antigen niches from other T cells with less-stringent TCRs to the individual antigens. Experimental ablation of both Tregs and memory-like T cells most efficiently make all antigenic niche available to naive T cells, inducing the activation of a broad repertoire of naive T cells (Figure 1f). Apparently, the disturbance of the T-cell system by the less-stringent activation of naive T cells results in autoimmunity, and it is hoped to fully reveal this mechanism at the network and systems levels.

Paradoxically, Treg depletion may result in less efficient immune response to viral infection. The depletion of Foxp3^+^ T cells during herpes simplex virus infection resulted in an accelerated fatal infection with increased viral loads, and thus Tregs are thought to facilitate early protective responses to local viral infection by allowing a timely entry of immune cells into the infected tissue.^42^ A similar observation was obtained by an immunisation experiment, where Treg depletion decreased the efficiency of immunisation.^43^ Under the feedback control perspective, the depletion of the entire Foxp3^+^ population empties all the antigen niches for Tregs, and thus induces the conversion of memory-like T cells to Tregs, and the activation of naive T cells, in order to fill the niches. This will induce less-stringent immune reactions, and deprive immunological resources for virus-specific T cells, and thereby decrease the efficiency of antiviral immune response. These lead to a hypothesis that the depletion of virus-specific Foxp3^+^ T cells only after the proper induction of T-cell response (the resolution phase in Figures 1b and d) may augment and prolong antiviral response without detrimental effects.

### Genetic fate-mapping

Genetic fate-mapping experiments showed that Tregs maintained Foxp3 expression in a relatively stable manner while the expression of Foxp3 in original naive T cells tends to be transient.^37, 44^ These studies, however, dismissed the cellular stability of the population and the stability of antigen presentation. In fact, while Tregs are more ‘proliferative' *in vivo* because they incorporate more bromodeoxyuridine than non-Treg CD4^+^ T cells,^45, 46^ Tregs seem to be always dying at a faster rate than other T cells. A genetic ablation of TCRα abolished bromodeoxyuridine incorporation in Tregs and resulted in a gradual loss of Treg population, a half-life of which was 6 weeks.^36^ In addition, these Tregs lost the activated (that is, regulatory) phenotype, although Foxp3 expression was mostly maintained.^36^ Collectively, these evidence strongly suggest that a continuous proliferation, induced by tonic TCR signaling, is required to maintain the size of the Treg population.

Importantly, a fate-mapping study showed that there was a significant flow into the memory-like Foxp3^−^CD44^high^ cells from the T cells that once expressed Foxp3.^37^ Considering that memory-like T cells more efficiently generate Foxp3^+^ T cells than naive T cells,^22^ Tregs and memory-like T cells may be in a close relationship and change their phenotypes to each other, obtaining an equilibrium in each antigenic niche.

Accordingly, we hypothesise that individual Treg survive, as long as their cognitive antigen niches are available (Figure 1). This leads to the following experimentally testable hypotheses: (1) Foxp3 expression in original naive T cells may not sustain if their cognitive antigens are occupied by existing Tregs; (2) the removal of a set of antigens may eliminate a repertoire of Tregs; and (3) a completely new external antigen that cannot be recognised by potentially cross-reactive T cells may create a new antigenic niche and thus induce Foxp3 expression in Foxp3^−^ T cells. To address these questions, we need to understand the temporal dynamics of T-cell numbers and phenotypes, as in our feedback control perspective, and to establish a new experimental method to identify newly generated Tregs or induced Tregs.

### Treg subsets

tTregs can acquire a part of Th cell differentiation mechanisms in the periphery and thereby control corresponding Th cell response. *Tbx21*-deficient Foxp3^+^ T cells fail to suppress the Th1-type inflammation of a Foxp3 hypomorphic mutant, *scurfy* mice.^47^ Similarly, the Foxp3^+^ T cell-specific deletion of *Irf4*, which is required for Th2 differentiation, results in the dysregulation of Th2 response,^48^ and Foxp3^+^ T cell-specific deletion of a Th17 factor, *Stat3*, results in a Th17-mediated colitis. Notably, *Stat3*-deficient Tregs lack the expression of C-C motif chemokine receptor 6 and fail to migrate to the colon tissue.^49^ The deletion of peroxisome proliferator-activated receptor-γ resulted in a specific impairment of Tregs to localise in visceral adipose tissue.^50^ Upon immunisation, some Foxp3^+^ Tregs express Blimp1 and acquire a number of features that share with T follicular helper cells (T_FH_) and are called as follicular Tregs. A cell transfer experiment showed that follicular Tregs were derived from tTregs.^43^ These evidence are in favour of the lineage perspective, as bulk ‘naive' Tregs can undergo further differentiation into more specialised Tregs, acquiring effector molecules, such as IL-10, tissue localisation and specific transcription factors.

On the other hand, as discussed above, the feedback control perspective predicts that a Treg subset may immediately develop from tTregs if existing tTregs have a TCR repertoire that is cross reactive to the immunised peptide, and otherwise naive T cells are activated and differentiated into a set of Tregs and memory-like/effector T cells that are adapted to a cytokine milieu or an anatomical location (for example, follicular Tregs and T_FH_; Figures 1b and d).

At the molecular level, T cells may activate multiple differentiation programmes irrespective of Foxp3 expression, which is compatible with the findings above.^47, 48, 49, 50^ For example, the transcriptional regulation of *Tbx21*, *Irf4* and *Stat3* seem independent from Foxp3 expression.^47, 48, 49^ This Foxp3-independent control of effector differentiation programmes may be especially important for enhancing the outputs of the memory-type control of T-cell activation (Figures 1d and e), which is considered inherently rapid but short by the preexisting negative and positive regulators. When multiple differentiation programmes need to be considered at the genomic level, it is essential to employ a multidimensional approach, and also, visualisation is critically important, as we discussed elsewhere.^51, 52^

### Molecular mechanism of Foxp3

Foxp3 has been regarded as a ‘master control gene' of Tregs in the lineage perspective. However, accumulating evidence suggest that Foxp3 is involved in, and a part of, the T-cell activation mechanism. New Foxp3 transcription occurs in activated T cells in the presence of IL-2 and TGF-β.^37, 53^ Although TGF-β is produced by various cells *in vivo*,^54^ IL-2 is produced mainly by CD44^high^ memory-like and activated T cells in the periphery.^55, 56^ Furthermore, the phenotype of Tregs, which is uniquely similar to activated T cells, is in fact established by strong TCR signal in the thymus^25, 26, 57^ and maintained by tonic TCR signal in the periphery.^36^ In addition, a DNase I hypersensitive site (DHS) sequencing showed that ‘Treg-specific' DHS were mostly independent from Foxp3 expression and were in fact those of activated T cells, while a chromatin immunoprecipitation sequencing showed that Foxp3 bound to these TCR-responsive enhancers.^58^ Meanwhile, it has been suggested that Foxp3 can bind to various transcription factors that are used for effector function in conventional T cells and thereby use them for their regulatory function.^4, 48, 49, 50, 58, 59^ Yet, importantly, these Foxp3-binding factors are mostly engaged in T-cell-activation-related events, including cytokine production and effector/helper T-cell differentiation. On the other hand, TCR signal is continuously delivered in Tregs,^24^ presumably because of their high-affinity TCRs to self and previously recognised antigens, and thereby sustains their activated phenotype and survival.^36^ Thus we propose a model that Foxp3 protein is expressed as a consequence of strong TCR signal and participates and modifies the TCR-induced transcriptional mechanism, where Foxp3-binding proteins such as nuclear factor of activated T-cells and Runx have key roles.

Interestingly, in humans, FOXP3 expression is transiently induced in any activated T cells by simple TCR stimulation.^60^ Although human FOXP3^high^ T cells are as suppressive as murine Foxp3^+^ T cells, activated T cells with transient FOXP3 expression are not suppressive on other T cells in *in vitro* assays and are thought to constitute the FOXP3^low^CD45RA^−^ non-Treg population that produces effector cytokines.^61^ FOXP3^low^ activated T cells show a lower, yet significant and equivalent level of FOXP3 expression as a suppressive, naive Treg population (that is, FOXP3^low^CD45RA^+^ T cells).^61^ Thus, at the molecular level, the transiently expressed FOXP3 may act as a negative regulator of T-cell activation in any activated T cells: activated T cells produce IL-2, which binds to and activates IL-2R on themselves in an autocrine manner, and thereby induces and prolongs the FOXP3 expression, while the expressed FOXP3 suppresses IL-2 production^62^ (Figure 1g). In fact, using *Foxp3-null* T-cell clone and FOXP3 knockdown in conventional T cells, McMurchy *et al.*^63^ showed that FOXP3 suppressed the proliferation and cytokine production in activated conventional T cells.

Collectively, FOXP3 regulates T-cell activation by both Treg-mediated (Figures 1c and e) and non-mediated (Figure 1g) mechanisms in humans, while Foxp3 mainly uses the Treg-mediated one in mice. Importantly, whether Treg-mediated or not, and whether in humans or in mice, Foxp3 expression occurs as a consequence of T-cell activation and regulates the T-cell activation via negative feedback control.

### Re-interpretation of neonatal thymectomy by the feedback control perspective

Given the widespread belief that Tregs are depleted by neonatal thymectomy,^9^ there has been confusion over the kinetics of Treg recovery after thymectomy. The number of CD4 T cells is dramatically decreased by neonatal thymectomy, in line with the level of immunosuppression induced by the treatment. The reduction is most striking in CD45RB^high^ naive T cells (~16-fold change) and is less so in CD25^+^CD4^+^ T cells (3–4-fold change) in 2–3 months old, day-3 thymectomised mice, which have relatively higher percentages of CD25^+^ or Foxp3^+^ Tregs.^14, 15, 16^ These CD25^+^ Tregs are as ‘regulatory' as those from non-treated wild-type mice in suppressing the development of colitis by cell transfer of CD45RB^high^ T cells into *Rag2*^*−/−*^ mice.^14^

Asano *et al.*^9^ found that CD25^+^CD4^+^ but not CD25^−^CD4^+^ cells suppress the development of autoimmune diseases caused by neonatal thymectomy. This is often regarded as evidence of the role of Tregs in thymectomy-induced autoimmunity.^2^ However, as neonatal thymectomy does not deplete Tregs, the suppression of autoimmune disease by transfer of Tregs does not imply that it is lack of Tregs that causes the autoimmune disease. Thus the mechanism of autoimmunity in thymectomised mice is yet to be revealed.

In the feedback control perspective, neonatal thymectomy diminishes the number of T cells within the TCR repertoire and thereby leaves many antigen niches unoccupied and available. The homeostatic pressure in the lymphopenic environment would induce the proliferation of a broad range of the naive T-cell repertoire that occupy those unoccupied niches through their low-yet-significant affinity TCR to self-antigens. Mature CD4^+^ T cells, especially self-reactive CD25^+^CD4^+^ T cells can prevent the development of autoimmune disease because they can fill the niche efficiently. The conclusive experiments to test this hypothesis would be to reveal the dynamics and TCR repertoire of T cells at a single cell level after thymectomy on various ages of mice, using a combined approach of experiments and mathematical modelling.

## Conclusions

Here we suggest that the fatal assumption in the study of suppressor T cells, which had collapsed the field, was that the suppressive activity was retained in a specialised cell or a single molecule. Although Tregs and Foxp3 act as negative regulators, immune regulation should be understood as a systems behavior. The proposed feedback control perspective will provide dynamical systems views to the modern Treg biology, relating new molecular and systems data to classical evidence.

Although the lineage perspective has been useful for analysing static relationships between cells and molecules, the proposed feedback control perspective is required to fully understand the dynamics of the T-cell system (Table 1) and will open up four important future directions in the Treg research area. First, the proposed perspective encourages the investigation of the dynamics of gene expression, cells, and even epigenetics, at single cell level. In fact, demethylation can occur within a few hours in some situations,^64^ and dynamical control is important in all these molecular and cellular events. Second, the proposed perspective is concerned with the dynamics rather than the stability of lineages and hence compatible with the study of systems-level dynamics based on mathematical modelling and dynamical systems and control theory, which will provide wider and deeper views on the control mechanisms of the T-cell system.^52^ Third, the proposed perspective provides a view on how the interaction of TCR repertoire and antigens from the T cells can induce different types of immune response. This will lead to a new dimension of T-cell biology, where the mechanisms of immunological tolerance and memory are revealed at TCR and antigen repertoire levels. Finally, the proposed perspective can be further extended to address other negative regulatory mechanisms in the T-cell system. For example, CTLA-4 (cytotoxic T-lymphocyte-associated protein 4) is inducible by TCR signaling and negatively regulates the T-cell activation process by inhibiting the interaction between CD28 and CD80/86 on APC,^65^ which provides another regulatory layer to the control mechanism of T-cell activation. Interestingly, CTLA-4 is also highly expressed in both activated T cells and Tregs.^65^ Thus the immunoregulatory T phenotype may be generalised as a stage of T-cell activation at which negative feedback mechanisms are actively operating.

## Figures and Tables

**Table 1:** Differences of the current dogma and the proposed perspective

	*Lineage perspective (current dogma)*	*Feedback control perspective (proposed)*
Foxp3	Lineage marker; master control gene	One among other key proteins in the T-cell activation control system
Thymic and peripheral Tregs	Fundamentally different classification	Different TCR repertoires that respond to either thymic or peripheral antigens
Identification of Tregs	Stability and plasticity are concerned; relationships between populations matter	Kinetics of genes and proteins and that of cell numbers matter
The purpose of epigenetic study	To distinguish stable and unstable Tregs	To analyse the dynamics of epigenetic modification and its mechanism
Experimental design	Comparison between Foxp3^+^ and Foxp3^−^ T cells	Time course analysis of Foxp3+, memory-like, activated and naive T cells
Data handling	Data have to be categorised and reduced so that distinct cell populations can be analysed	Aims to use original continuous and multidimensional data of gene expressions; compatible with single cell sequencing

Abbreviations: TCR, T-cell receptor; Treg, regulatory T cell.

## Box 1 Memory-like T cells

Memory-like, or memory phenotype, is a commonly used term to phenotypically define a T-cell population typically by the following markers: CD44^high^CD45RB^low^Foxp3^−^ CD25^−^. Although this population does not include Tregs and naive T cells and contains antigen-experienced memory T cells, the memory-like T cells, as a population, may have different properties to individual antigen-experienced memory T cells, which are produced by immunisation or infection in an antigen-specific manner, especially in their proliferative activity *in vivo*.^66^
